# Schisandrol B and schisandrin B inhibit TGFβ1-mediated NF-κB activation via a Smad-independent mechanism

**DOI:** 10.18632/oncotarget.23213

**Published:** 2017-12-14

**Authors:** Jung Nyeo Chun, Soonbum Park, Sanghoon Lee, Jae-Kyung Kim, Eun-Jung Park, MinJi Kang, Hye Kyung Kim, Jong Kwan Park, Insuk So, Ju-Hong Jeon

**Affiliations:** ^1^ Department of Physiology and Biomedical Sciences, Seoul National University College of Medicine, Seoul 03080, Korea; ^2^ Institute of Human-Environment Interface Biology, Seoul National University, Seoul 03080, Korea; ^3^ Department of Biochemistry, University of Utah School of Medicine, Salt Lake City, UT 84112-5650, USA; ^4^ College of Pharmacy, Kyungsung University, Busan 48434, Korea; ^5^ Department of Urology, Medical School, and Institute for Medical Sciences, Chonbuk National University, Jeonju 54896, Korea

**Keywords:** schisandra chinensis, schisandrol B, schisandrin B, TGFβ1, NF-κB

## Abstract

Aberrant transforming growth factor β1 (TGFβ1) signaling plays a pathogenic role in the development of vascular fibrosis. We have reported that *Schisandra chinensis* fruit extract (SCE), which has been used as a traditional oriental medicine, suppresses TGFβ1-mediated phenotypes in vascular smooth muscle cells (VSMCs). However, it is still largely unknown about the pharmacologic effects of SCE on various TGFβ1 signaling components. In this study, we found that SCE attenuated TGFβ1-induced NF-κB activation and nuclear translocation in VSMCs. Among the five active ingredients of SCE that were examined, schisandrol B (SolB) and schisandrin B (SchB) most potently suppressed TGFβ1-mediated NF-κB activation. In addition, SolB and SchB effectively inhibited IKKα/β activation and IκBα phosphorylation in TGFβ1-treated VSMCs. The pharmacologic effects of SolB and SchB on NF-κB activation were independent of the Smad-mediated canonical pathway. Therefore, our study demonstrates that SCE and its active constituents SolB and SchB suppress TGFβ1-mediated NF-κB signaling pathway in a Smad-independent mechanism. Our results may help further investigations to develop novel multi-targeted therapeutic strategies that treat or prevent vascular fibrotic diseases.

## INTRODUCTION

Transforming growth factor β1 (TGFβ1) mediates tissue repair or wound healing processes by regulating various molecular and cellular mechanisms, including cell migration, proliferation, and extracellular matrix (ECM) production [1, 2]. However, aberrant TGFβ1 signaling disturbs physiological tissue remodeling, which leads to pathologic fibrotic changes [3]. Particularly, TGFβ1 is involved in the pathogenesis of a range of vascular fibrotic diseases, such as restenosis, atherosclerosis, and hypertension [4–6]. In these pathological states, TGFβ1 acts on vascular smooth muscle cells (VSMCs) to induce synthetic phenotypes, including cell migration and proliferation, to the injured sites [7–10].

NF-κB is a transcription factor that controls the expression of genes involved in various biological processes, including inflammation and cell survival [11–14]. Deregulated activation of NF-κB is closely associated with many diseases, including cancer and vascular human diseases [11, 15–18]. In VSMCs, NF-κB participates in the progress of vascular fibrotic diseases via multiple cellular processes, including increased cell migration and neointima formation [19, 20]. In addition, NF-κB activity is elevated during the normal aging process, which contributes to the development of vascular diseases [21, 22]. Therefore, NF-κB signaling pathway has gained attention as a promising a therapeutic target for treatment of vascular fibrosis [23–25].

TGFβ1 engagement of the type II receptor (TβRII) serine/threonine kinases at the plasma membrane allows TβRII to phosphorylate TβRI [26, 27]. In turn, the activated TβRI propagates the signals through both the Smad-dependent canonical pathways and the Smad-independent non-canonical pathways [28–30]. Therefore, the cellular output to TGFβ1 signaling is influenced by interaction between canonical and non-canonical signaling cascades. Accumulating evidence has shown that TGFβ1 activates NF-κB pathway via the non-canonical pathways [28, 31], suggesting that TGFβ1-NF-κB signaling axis plays a crucial role in the pathogenesis of vascular fibrotic diseases.

*Schisandra chinensis* fruit extract (SCE) has been used as a traditional oriental medicine and shown to be effective in the treatment of cardiovascular diseases [32]. We have demonstrated that SCE and its active ingredient schisandrin B (SchB) effectively inhibit TGFβ1-induced Smad activation and myosin light chain (MLC) phosphorylation in VSMCs [33, 34]. These results suggest that SCE or its active components can be used as multi-targeted therapeutic agents that attenuate or prevent vascular fibrotic diseases.

In this study, we investigated the effect of SCE and its active ingredients on TGFβ1-NF-κB signaling axis in A7r5 VSMCs. We discovered that SCE inhibited TGFβ1-induced NF-κB activation. Of the five active ingredients of SCE that were examined, schisandrol B (SolB) and SchB were most potently inhibited TGFβ1-NF-κB signaling axis via a Smad-independent mechanism. Our results provide insight into understanding the molecular mechanisms of pharmacologic actions of SCE and its active constituents on vascular fibrosis.

## RESULTS

### SCE inhibits TGFβ1-induced NF-κB activation in A7r5 cells

**Figure 1:**
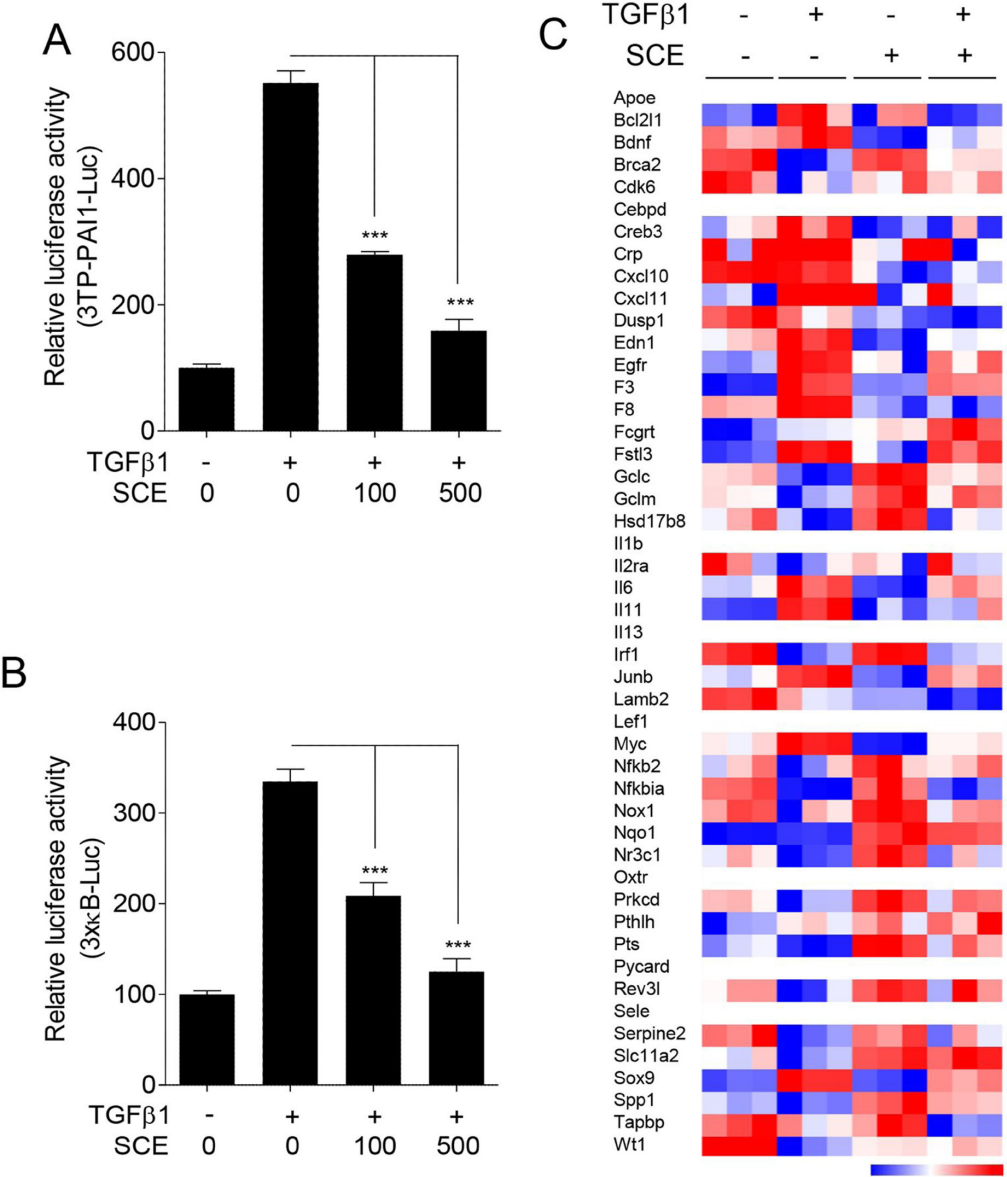
SCE inhibits TGFβ1-induced NF-κB activation in A7r5 cells The cells were transfected with 3TP-PAI1-Luc (**A**) or 3×kB-Luc (**B**) reporter constructs and then treated with TGFβ1 (1 ng/ml) and/or SCE (100 or 500 mg/ml) for 24 h. The luciferase activity was expressed as a relative value compared to that of the untreated cells which was set to 100%. The data were expressed as the mean ± SEM (*n* = 3–5). ^***^p < 0.005. (**C**) The heatmap shows SCE-regulated NF-κB target genes in TGFβ-treated cells.

It has been known that SCE suppresses TGFβ1 signaling in fibrotic responses [33, 34]. On the other hand, it has been found that SCE inhibits NF-κB signaling in inflammatory responses [35–37]. Based on these findings, we have raised a question whether SCE inhibits TGFβ1-induced NF-κB activation in VSMCs. To solve this question, we first performed luciferase assays using reporter gene constructs containing Smad- or NF-κB-binding elements in TGFβ1-treated A7r5 cells. As expected [33], SCE inhibited Smad-mediated luciferase activity (Figure 1A). Similarly, SCE suppressed NF-κB-mediated luciferase activity in a dose-dependent manner (Figure 1B), indicating that SCE inhibits TGFβ1-induced NF-κB activation.

To confirm these results, we analyzed the microarray data (GSE87439) obtained from A7r5 cells treated with TGFβ1 and/or SCE. The SAM analysis identified that TGFβ1 induces changes in the expression levels of 3840 genes in A7r5 cells. Of the 3840 genes, SCE completely or partially reversed the expression levels of 2147 genes in TGFβ1-treated cells (data not shown). We also found that TGFβ1 affects the expression levels of 98 NF-κB target genes in A7r5 cells. Of the 98 genes, SCE completely or partially reversed the expression levels of 48 genes in TGFβ1-treated cells. The quantitative graphs showed the typical NF-κB target genes expression induced by TGFβ1 which regulated by SCE. These results demonstrate that SCE inhibits TGFβ1-induced NF-κB activation in addition to Smad (Figure 1C and Supplementary Figure 1).

### SCE inhibits TGFβ1-induced IKK activation and IκBα degradation in A7r5 cells

**Figure 2:**
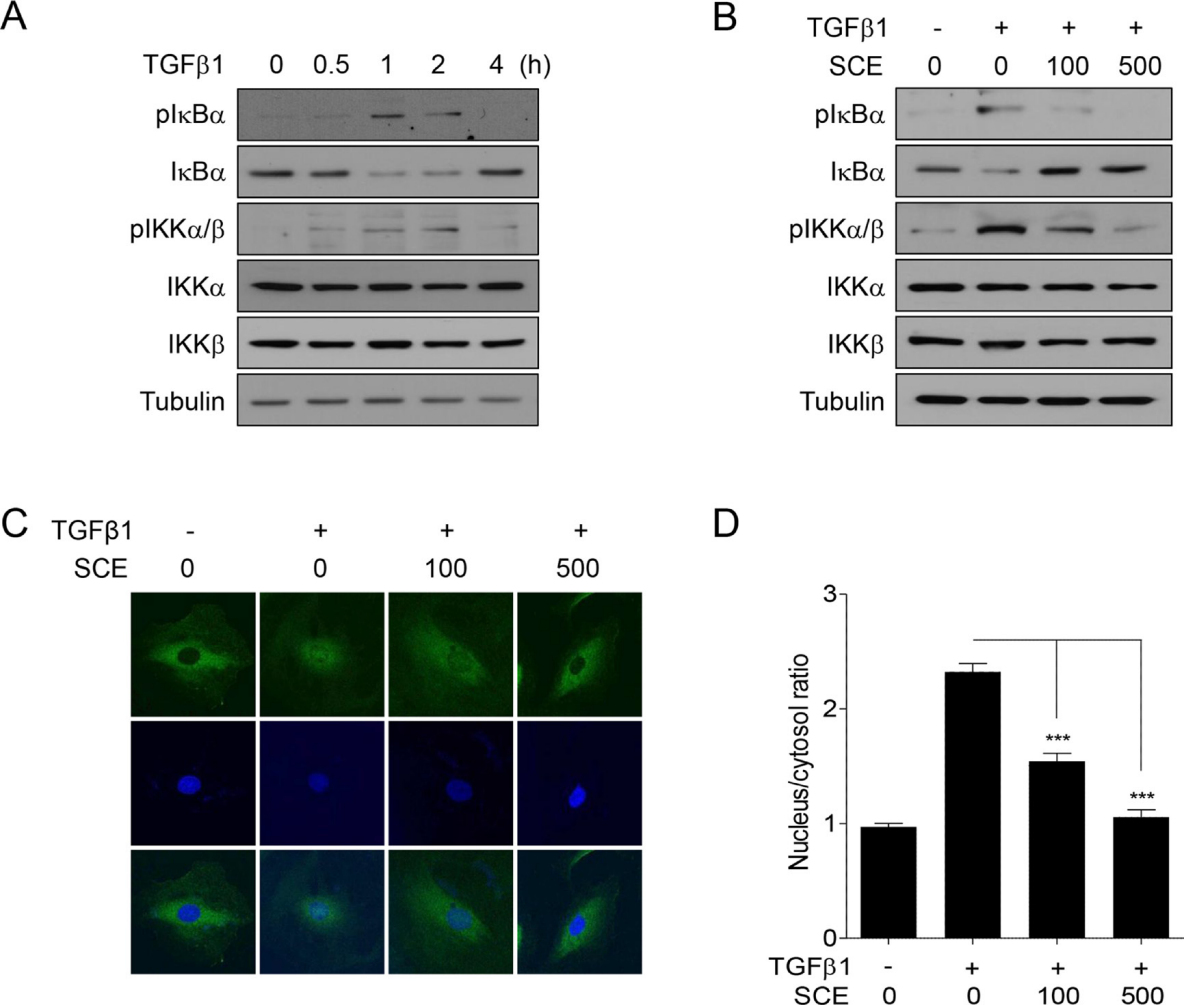
SCE inhibits TGFβ1-induced IKK activation and IκBα degradation in A7r5 cells The cells were treated with TGFβ1 (1 ng/ml) and/or SCE (100 or 500 mg/ml) for the indicated times (**A**) or for 1 h (**B**) prior to western blot analysis. (**C**) The cells were treated with TGFβ1 (1 ng/ml) and/or SCE (100 or 500 mg/ml) for 1 h prior to confocal microscopy. The subcellular localization of p65 was assessed using anti-p65 antibody and FITC-conjugated IgG antibody. DAPI was used to visualize the nucleus. (**D**) The nuclear/cytosolic ratio of p65 was measured in at least 15 independent fields (*n* = 4). The data were expressed as the mean ± SEM. ^***^p < 0.005.

To further confirm the inhibitory effect of SCE on TGFβ1-induced NF-κB activation, we examined whether SCE affect IKK signaling pathway. Western blot analysis showed that TGFβ1 increased the levels of phospho-IκBα and -IKKα/β and concomitantly decreased those of total IκBα following 1 h of treatment with TGFβ1 (Figure 2A). Under the same condition, SCsE markedly suppressed TGFβ1-mediated phosphorylation of IκBα and IKKα/β and degradation of IκBα (Figure 2B). In addition, confocal microscopic analysis revealed that SCE inhibited TGFβ1-induced NF-κB translocation to the nucleus (Figure 2C and 2D). Therefore, these results demonstrate that SCE inhibits TGFβ1-induced IKK activation and IκBα degradation.

### SolB and SchB inhibit TGFβ1-induced NF-κB activation in A7r5 cells

**Figure 3:**
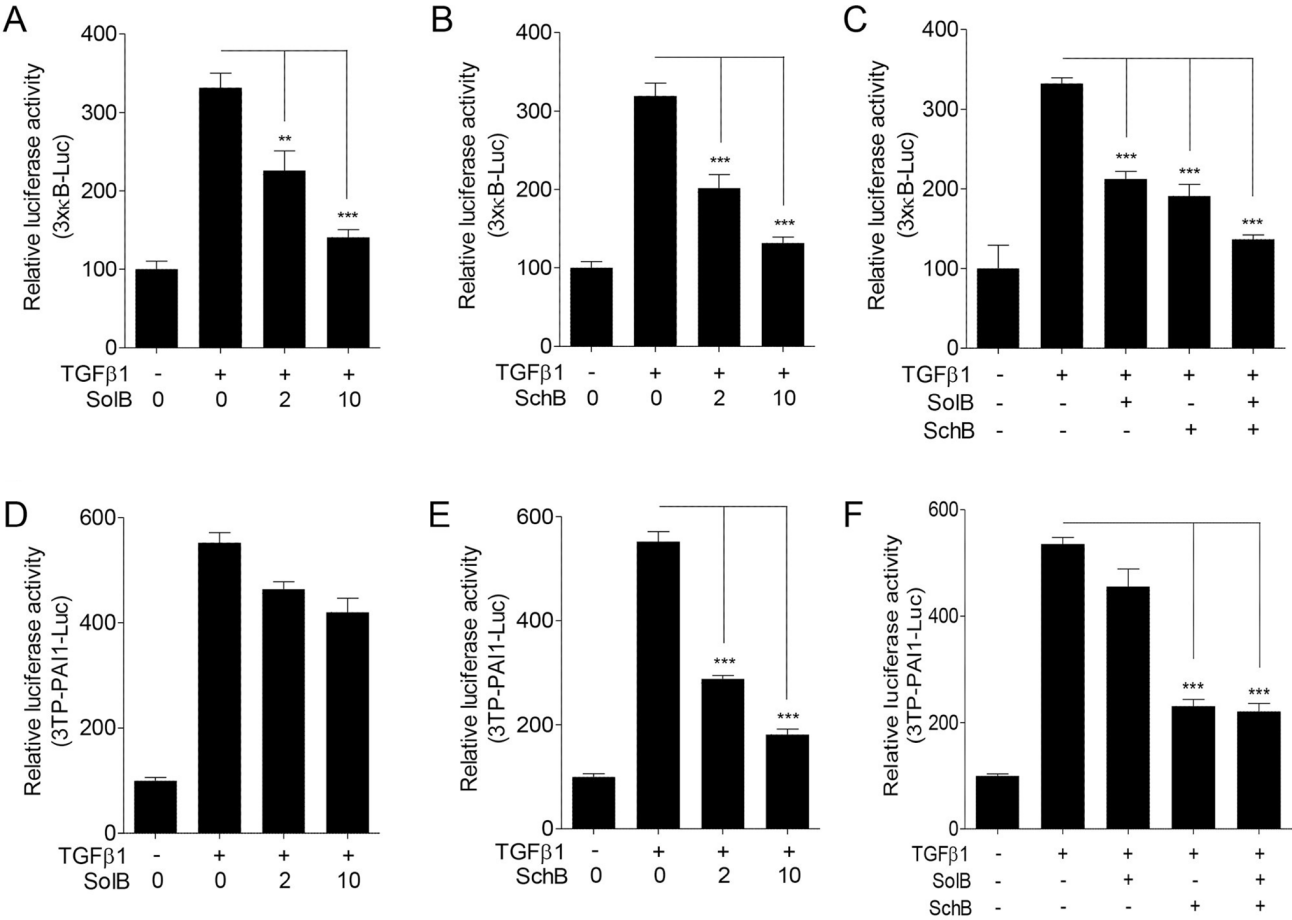
SolB and SchB inhibit TGFβ1-induced NF-κB activation in A7r5 cells The cells were transfected with 3×kB-Luc (**A–C**) or 3TP-PAI1-Luc (**D–F**) reporter constructs and then treated with TGFβ1 (1 ng/ml) and/or SolB (2 or 10 µM) or SchB (2 or 10 µM) for 24 h. The luciferase activity was expressed as a relative value compared to that of the untreated cells which was set to 100%. The data were expressed as the mean ± SEM (*n* = 4). ^**^p < 0.01, ^***^p < 0.005.

To identify the effective ingredients of SCE against TGFβ1-induced NF-κB activation, we examined five active ingredients of SCE using luciferase assays. Among these compounds, schisandrol B (SolB) and schisandrin B (SchB) most potently inhibited NF-κB activity in TGFβ1-treated A7r5 cells, whereas schisandrin C slightly reduced NF-κB activity (Supplementary Figure 2). SolB and SchB suppressed NF-κB-mediated luciferase activity in a dose-dependent manner (Figure 3A and 3B). Therefore, we chose SolB and SchB as effective components for the following studies. SolB and SchB co-treatment showed additive inhibitory effect on NF-κB activity (Figure 3C). Interestingly, we found that SolB and SchB exert different pharmacologic effects in TGFβ1-treated A7r5 cells. SolB inhibited NF-κB activity, whereas it did not affect Smad activity (Figure 3D). On the other hand, SchB suppressed both NF-κB and Smad activity (Figure 3E). Co-treatment of SolB and SchB was not shown any additive effects on Smad activity (Figure 3F).

**Figure 4:**
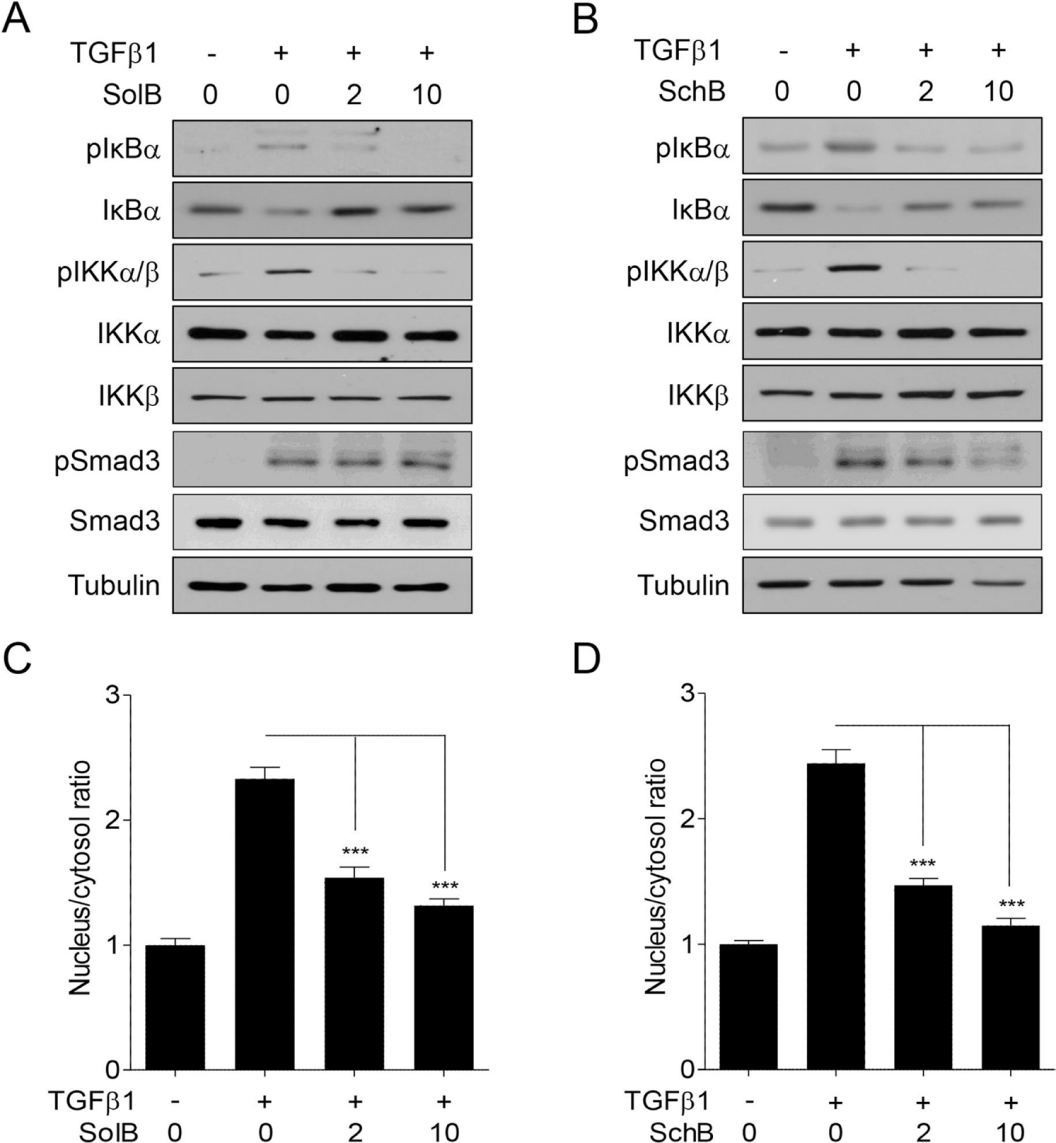
SolB and SchB inhibit TGFβ1-induced IKK activation and IκBα degradation in A7r5 cells The cells were treated with TGFβ1 (1 ng/ml) and/or SolB (2 or 10 µM) or SchB (2 or 10 µM) for 1 h prior to western blot analysis (**A**, **B**) or confocal microscopy (**C**, **D**). The nuclear/cytosolic ratio of p65 was measured in at least 15 independent fields (*n* = 4). The data were expressed as the mean ± SEM. ^***^p < 0.005.

To determine the molecular mechanisms of SolB and SchB action on NF-κB activity, we examined the phosphorylation level of IκBα and IKKα/β in A7r5 cells. TGFβ1 elevated the phosphorylation levels of IκBα and IKKα/β and concomitantly reduced those of total IκBα (Figure 4A and 4B). Under the same condition, SolB and SchB reversed these TGFβ1-induced molecular changes (Figure 4A and 4B). But the phosphorylation of Smad3 was not reversed by SolB (Figure 4A). These results were further confirmed by confocal microscopic analysis. As presented in Figure 4C and 4D, SolB and SchB inhibited TGFβ1-induced nuclear translocation of NF-κB in A7r5 cells.

**Figure 5:**
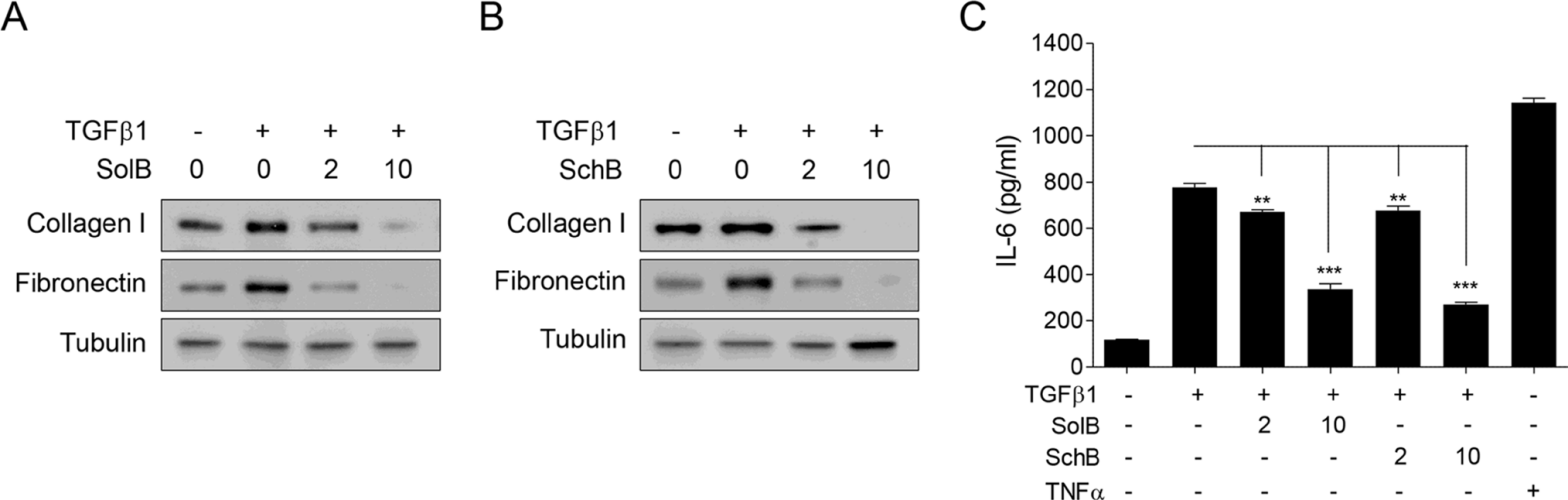
SolB and SchB inhibit TGFβ1-induced NF-κB target gene production in A7r5 cells The cells were stimulated with TGFβ1 (1 ng/ml) and/or SolB (2 or 10 µM) or SchB (2 or 10 µM) for 48 h prior to western blot analysis (**A–C**). (C) For IL-6 measurement, the medium was collected at 48 h after treatment. TNFα (10 ng/ml) used as a positive control for NF-κB activation. IL-6 level was determined by ELISA assay kit according to the manufacturer’s instruction. The data were expressed as the mean ± SEM (*n* = 4). ^**^p < 0.01, ^***^p < 0.005.

To test whether SolB and SchB could affect the production of NF-κB-regulated profibrotic gene expression and -cytokine, we checked expression of collagen I, fibronectin, and secretion of Interleukin (IL)-6. SolB and SchB inhibited TGFβ1-induced expression of collagen I and fibronectin in dose-dependent manner (Figure 5A and 5B). And SolB and SchB were significantly decreased of the secretion of IL-6 (Figure 5C). These results demonstrate that SolB and SchB inhibit TGFβ1-induced NF-κB activation and its target gene expression.

### Smad activity is irrelevant to NF-κB activity in TGFβ1-treated A7r5 cells

**Figure 6:**
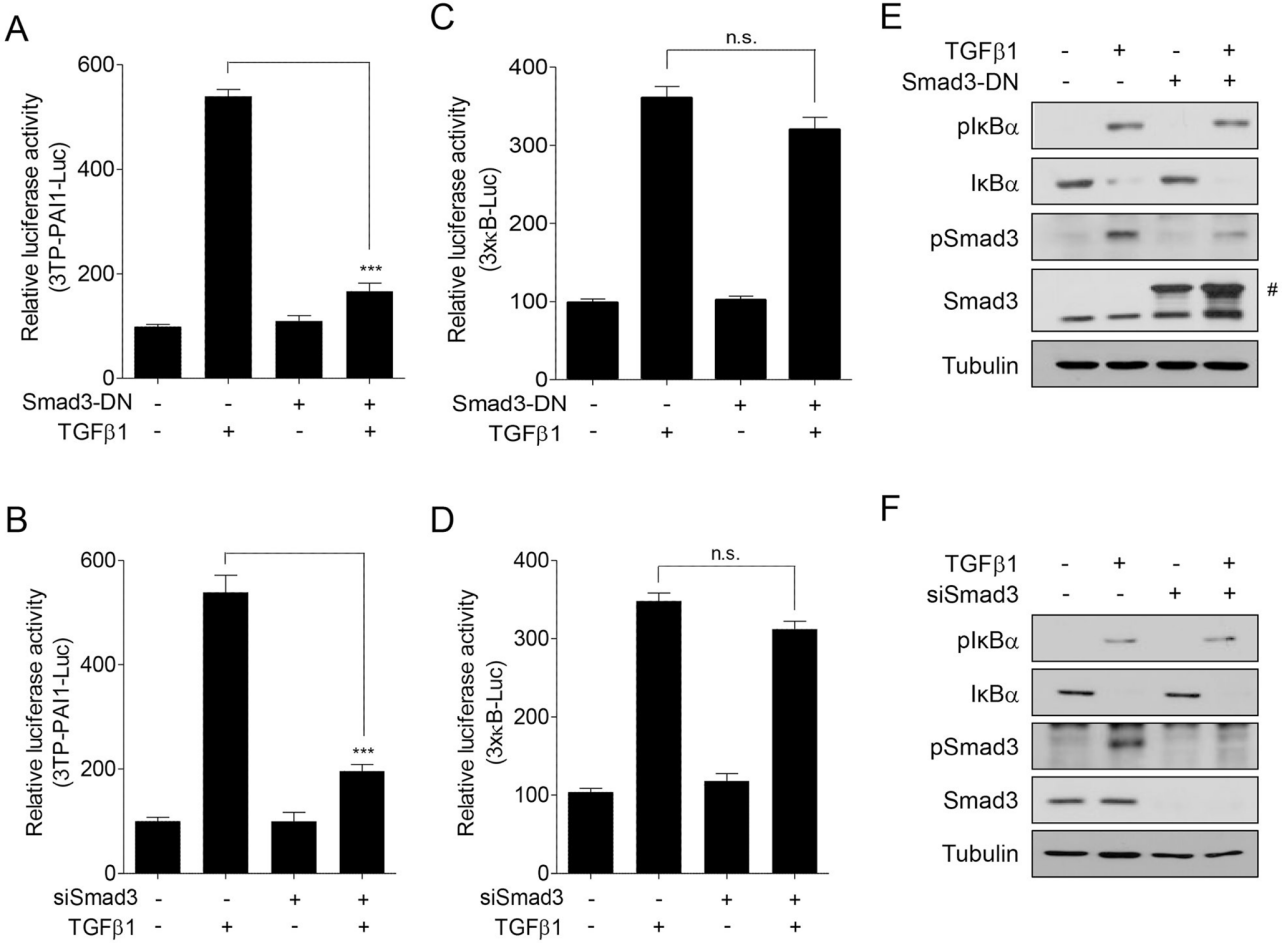
Smad activity is irrelevant to NF-κB activity in TGFβ1-treated A7r5 cells The cells were transfected with 3TP-PAI1-Luc (**A**, **B**) or 3×kB-Luc (**C**, **D**) reporter constructs. Under the condition, the cells were co-transfected with Smad3-DN (A and C) or siSmad3 (B and D). The transfected cells were further treated with TGFβ1 (1 ng/ml) for 24 h. The luciferase activity was expressed as a relative value compared to that of the untreated cells which was set to 100%. The data were expressed as the mean ± SEM (*n* = 4). ^***^p < 0.005. n.s., not significant. (**E**, **F**) The cells were transfected with Smad3-DN or siSMAD3 for 48 h and then treated with TGFβ1 (1 ng/ml) for 1 h prior to western blot analysis. ^#^GFP-Smad3.

We found that SolB and SchB have different pharmacologic effects on Smad and NF-κB activity (Figure 3). Based on these observations, we investigated whether Smad activity affects NF-κB activity in TGFβ1-treated cells. We first examined the effect of Smad3-DN or siSmad3 on NF-κB activity. Both Smad3-DN and siSmad3 inhibited Smad-mediated luciferase activity in TGFβ1-treated cells (Figure 6A and 6B). In contrast, they did not affect NF-κB-mediated luciferase activity (Figure 6C and 6D). In addition, they did not influence on the levels of phospho- and total IκBα (Figure 6E and 6F). These results indicate that TGFβ1-induced NF-κB activation is independent of Smad activity. In addition, our findings suggest that SolB and SchB suppress TGFβ1-induced NF-κB activation by inhibiting Smad-independent IKK pathway.

## DISCUSSION

Aberrant regulation of TGFβ1 signaling underlies the pathogenesis of vascular fibrotic diseases, including atherosclerosis and restenosis. We have reported that SCE and its active ingredient SchB inhibit TGFβ1-induced Smad activation and myosin light chain phosphorylation [33, 34]. In this study, we found that SCE inhibits TGFβ1-induced NF-κB activation. Among the active ingredients of SCE tested, SolB and SchB most potently suppressed TGFβ1-induced NF-κB activation by inhibiting the Smad-independent IKK pathway. Our study broadens understanding of the molecular mechanisms by which SCE and its active ingredients inhibit TGFβ1-induced phenotypes of VSMCs.

TGFβ1 controls a variety of signaling molecules via the Smad-dependent or -independent mechanisms. We have shown that SCE and its ingredients exert their pharmacologic effects by inhibiting canonical and non-canonical pathways of TGFβ1 signaling [33, 34]. Interestingly, SolB and SchB have different pharmacologic activity on TGFβ1 signaling Figure 3). SolB inhibited TGFβ1-induced NF-κB activation, but not Smad. In contrast, SchB suppressed both Smad and NF-κB in TGFβ1 signaling pathways. These results indicate that NF-κB activity does not affect Smad activity in TGFβ1-treated cells. In addition, we demonstrated that Smad does not affect TGFβ1-mediated NF-κB activity (Figure 6). Therefore, our findings demonstrate that Smad and NF-κB are unrelated to each other in TGFβ1-treated VSMCs and that SCE and its constituents inhibit canonical and non-canonical signaling of TGFβ1 via a separate mechanism.

NF-κB plays a pleiotropic role in a range of cellular processes, such as cell survival, proliferation, inflammation, and cell invasion, in response to various extracellular stimuli [11, 12, 38]. Therefore, NF-κB has been considered as a promising a therapeutic target for treatment of cancer and inflammatory diseases [11, 20, 39–41]. Particularly, NF-κB has been known to mediate pathogenic functions in the development of fibrotic diseases [17, 42]. Here, we found that SCE and its ingredients suppress NF-κB activity by inhibiting IKK activation and thereby IκBα phosphorylation and NF-κB nuclear translocation. These results suggest that SCE and its active constituents may be useful to treat a range of NF-κB-mediated diseases.

In summary, the present study demonstrated that SCE and its ingredients SolB and SchB inhibit TGFβ1-induced NF-κB activation in VSMCs. Our results provide a scientific basis for future investigation aiming at understanding and treating TGFβ1-induced vascular fibrotic diseases.

## MATERIALS AND METHODS

### Cell culture and reagents

The A7r5 rat aortic smooth muscle cell line was obtained from ATCC (CRL –1444). Cells were cultured in DMEM supplemented with 10% fetal bovine serum, penicillin (100 U/ml), and streptomycin (100 μg/ml). Prior to treatment with TGFβ1 (R&D Systems, Minneapolis, MN), cells were maintained in DMEM containing 0.2% FBS for 2 h. All cell culture agents were purchased from Hyclone (Logan, UT) or Gibco (Grand Island, NY). SCE and its constituents were prepared as described in our papers [33, 34, 43]. All other reagents not specified were supplied by Sigma-Aldrich (St. Louis, MO).

### Microarray experiment and computational analysis

Microarray experiments were performed using the cells treated with 100 mg/ml SCE for 24 h as described in our previous papers [44–46]. The microarray data, which are available through the Gene Expression Omnibus (GEO) database (accession number GSE87439), were normalized using single-channel array normalization (SCAN) method, which is efficient to reduce array-specific background for standardization of individual probe-level data [45, 47]. Microarray probes were mapped to 13,877 genes using a custom mapping file, Rat2302_Rn_ENTREZG (version 19.0.0) which is provided by the BrainArray resource (http://brainarray.mbni.med.umich.edu/brainarray/). The Significance Analysis of Microarrays (SAM) analysis was carried out to identify differentially expressed genes (DEGs) among 314 NF-κB target genes (http://www.bu.edu/NF-κB/gene-resources/target-genes/). A tuning parameter, delta of 0.4, optimized the cutoff for significance with the estimation of false discovery rate (FDR) threshold *q*-value of 0.01.

### Transfection

Cells were transfected with 100 nM siRNA against Smad3 (siSmad3) for 48 h [48] using Lipofectamine RNAiMAX reagent (Invitrogen, Karlsruhe, Germany). The siRNAs were purchased from Qiagen (Hilden, Germany). Cells were also transfected with the dominant negative mutant of Smad3 (Smad3-DN) in pEGFP-N1 [49] using FuGENE 6 according to the manufacturer’s protocol (Roche, Mannheim, Germany)

### Luciferase assay

Cells were transfected with 3×κB-Luc [50] or 3TP-PAI1-Luc [33] reporter gene plasmids using FuGENE 6. At 24 h after transfection, the cells were incubated with TGFβ1, SCE, and/or its active ingredients for 24 h. The cells were harvested and assayed for luciferase activity using a commercial kit (Promega, Madison, WI). The luciferase activity was normalized to β-galactosidase activity.

### Western blot analysis

Antibodies against pIκBα^S32/36^, IκBα, pIKKα/β^S176/180^, IKKα/β, pSmad3^S423/425^, and Smad3 were obtained from Cell Signaling Technology (Beverly, MA). Anti-collagen I, anti-fibronectin, and anti-tubulin antibodies were purchased from abcam (Cambridge, UK), Santa Cruz Biotechnology (Santa Cruz, CA), and Sigma-Aldrich, respectively. The crude extracts were resolved in 6–10% SDS-PAGE gels and probed with the indicated antibodies. The data shown are representative of at least three independent experiments. Quantification for Western blots is shown in Supplementary Figure 3.

### Confocal microscopy

Cells were grown on glass coverslips in 12-well plates. After cells were treated with TGFβ1, SCE, SolB, and/or SchB for 1 h, the cells were fixed with 3.7% formaldehyde in PBS for 10 min, permeabilized with 0.1% Triton X-100 for 5 min, and blocked with 5% normal goat serum in PBS for 30 min. The cells were labeled with anti-p65/RelA antibody (Santa Cruz, CA) for overnight at 4°C and then probed with FITC-conjugated anti-rabbit IgG antibody (Invitrogen) and DAPI (Roche) for additional 1 h at RT. The cells were photographed using the FluoView 1000 confocal microscope (Olympus, Tokyo, Japan).

### Interleukin (IL)-6 measurements

A7r5 cells (1.2 × 10^4^) cultured in 12-well plates. Cells were treated with TGFβ1 with or without SolB or SchB for 24 h after then cells were starved in DMEM containing 0.2% FBS. Then the cultured medium collected. IL-6 concentrations were determined by ELISA assay kit according to the manufacturer’s instructions (R&D Systems).

### Statistical analysis

All data are expressed as mean ± SEM. Comparison of means among experimental groups was carried out with ANOVA followed by a post hoc test. *p* < 0.05 was considered statistically significant.

## SUPPLEMENTARY MATERIALS FIGURES


