# Supplementary material for: Schisandrol B and schisandrin B inhibit TGFβ1-mediated NF-κB activation via a Smad-independent mechanism
Source: Oncotarget. 2017 Dec 14;9(3):3121–30. doi: 10.18632/oncotarget.23213 (PMC5790451; doi:10.18632/oncotarget.23213)
Supplement: Supplementary file 1 [file oncotarget-09-3121-s001.pdf]

Schisandrol B and schisandrin B inhibit TGFβ1-mediated NF-κB activation via a Smad-independent mechanism

SUPPLEMENTARY MATERIALS

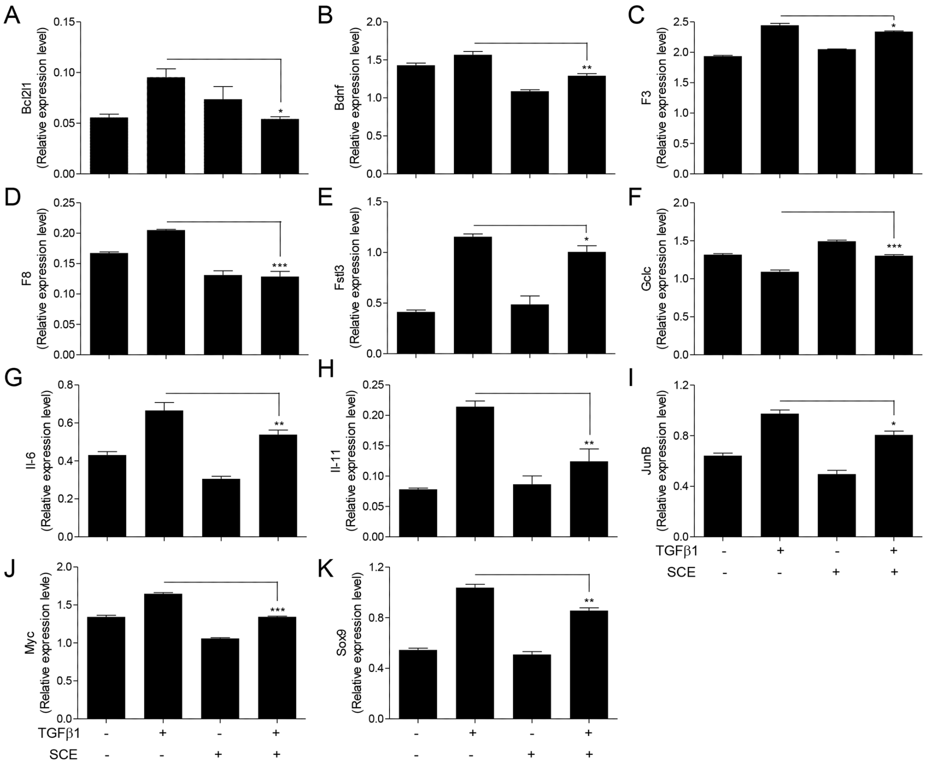

Supplementary Figure 1: The quantitative graph of 11 typical NF-κB target genes. The relative expression value of NF-κB target genes compared to that of the untreated cells was represented. (**A**) Bcl2l1, (**B**) Bdnf, (**C**) F3, (**D**) F8, (**E**) Fstl3, (**F**) Gclc, (**G**) Il-6, (**H**) Il-11, (**I**) JunB, (**J**) Myc, (**K**) Sox9. $^{*}p < 0.05$, $^{**}p < 0.01$, $^{***}p < 0.005$.

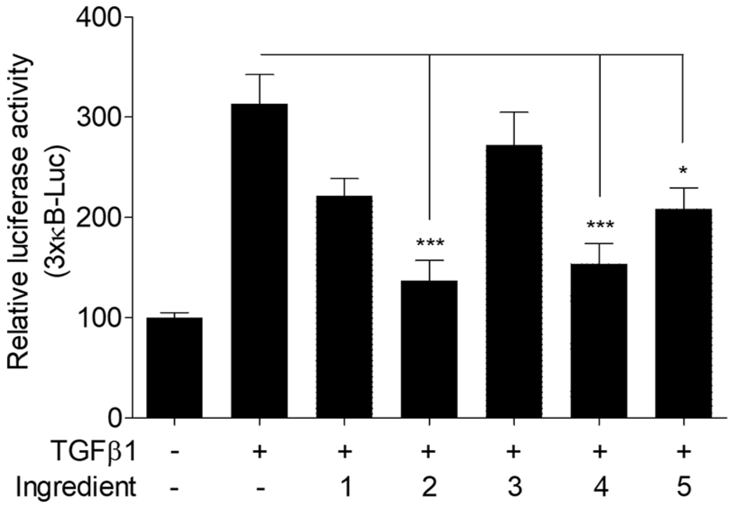

Supplementary Figure 2: The effect of 5 ingredients of *Schisandra chinensis* extract on TGFβ1-induced NF-κB activation in A7r5 cells. The cells were transfected with 3×kB-Luc reporter construct and then treated with TGFβ1 (1 ng/ml) and/or the indicated ingredients (10 μM each) for 24 h. The luciferase activity was expressed as a relative value compared to that of the untreated cells which was set to 100%. The data were expressed as the mean ± SEM ($n = 4$). $^{*}p < 0.05$, $^{***}p < 0.005$. Ingredient 1, Schisandrol A; 2, Schisandrol B; 3, Schisandrin A; 4, Schisandrin B; 5, Schisandrin C.

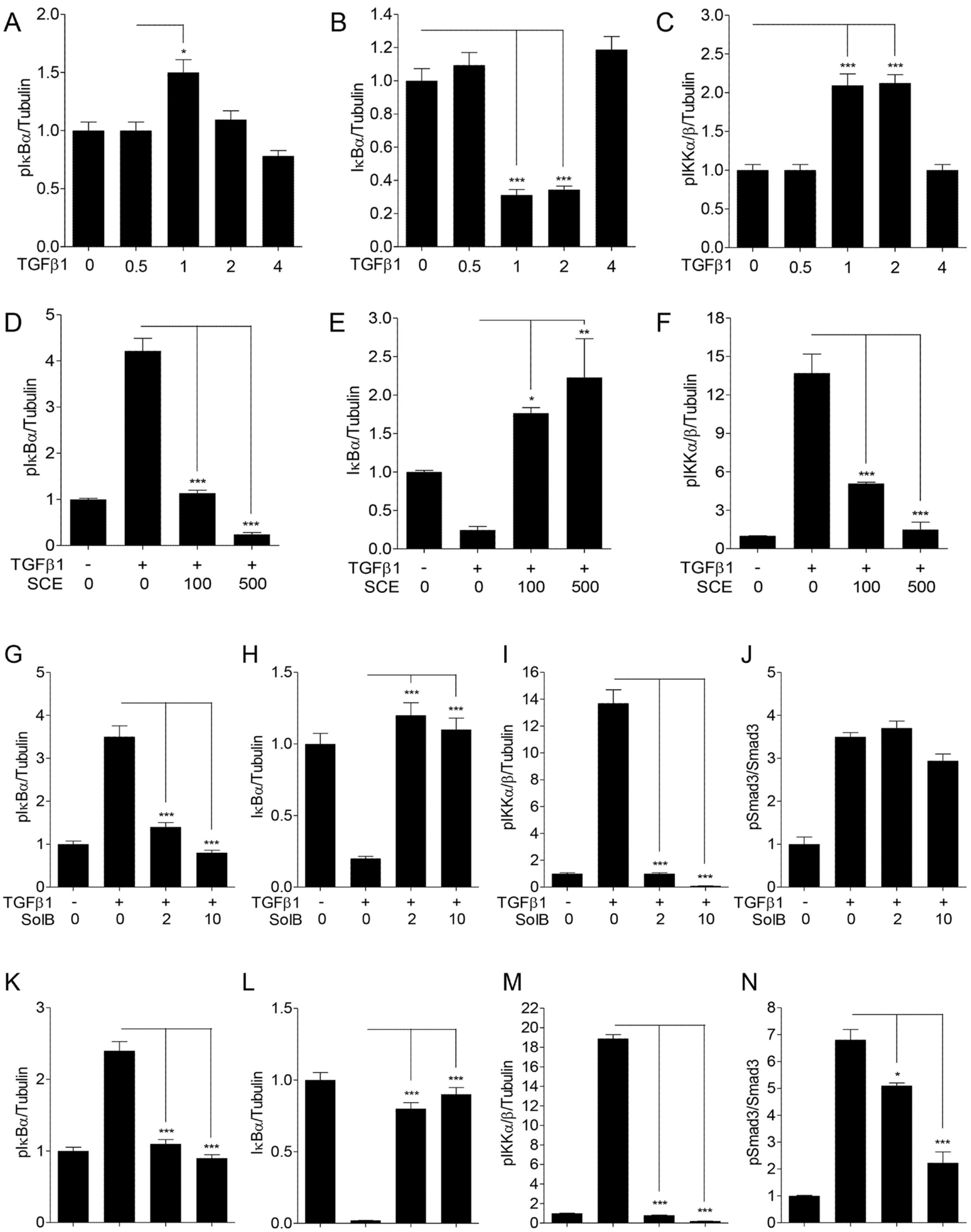
A
B
C
D
E
F
G
H
I
J
K
L
M
N
pIκBα/Tubulin
IκBα/Tubulin
pIKKα/β/Tubulin
pSmad3/Smad3
TGFβ1 0 0.5 1 2 4
TGFβ1 - + + +
SCE 0 0 100 500
SolB 0 0 2 10
*
**
***

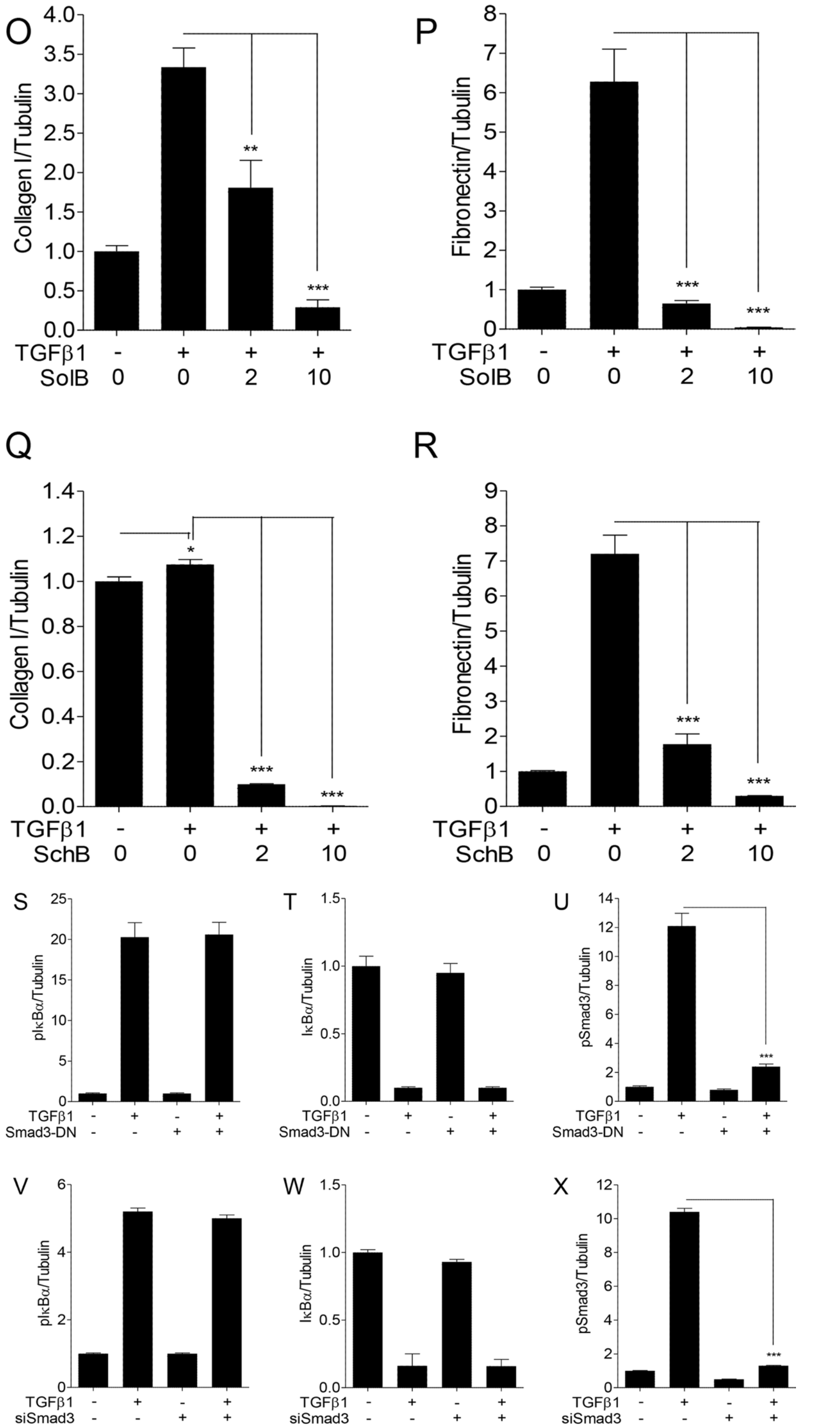

Supplementary Figure 3: Quantitative representation of western blots. Quantitative representations of Figure 2A (**A–C**), Figure 2B (**D–F**), Figure 4A (**G–J**), Figure 4B (**K–N**), Figure 5A (**O**, **P**), Figure 5B (**Q**, **R**), Figure 6E (**S–U**), and Figure 6F (**V–X**) are shown. The data were expressed as the mean ± SEM. $^{*}p < 0.05$, $^{**}p < 0.01$, $^{***}p < 0.005$.

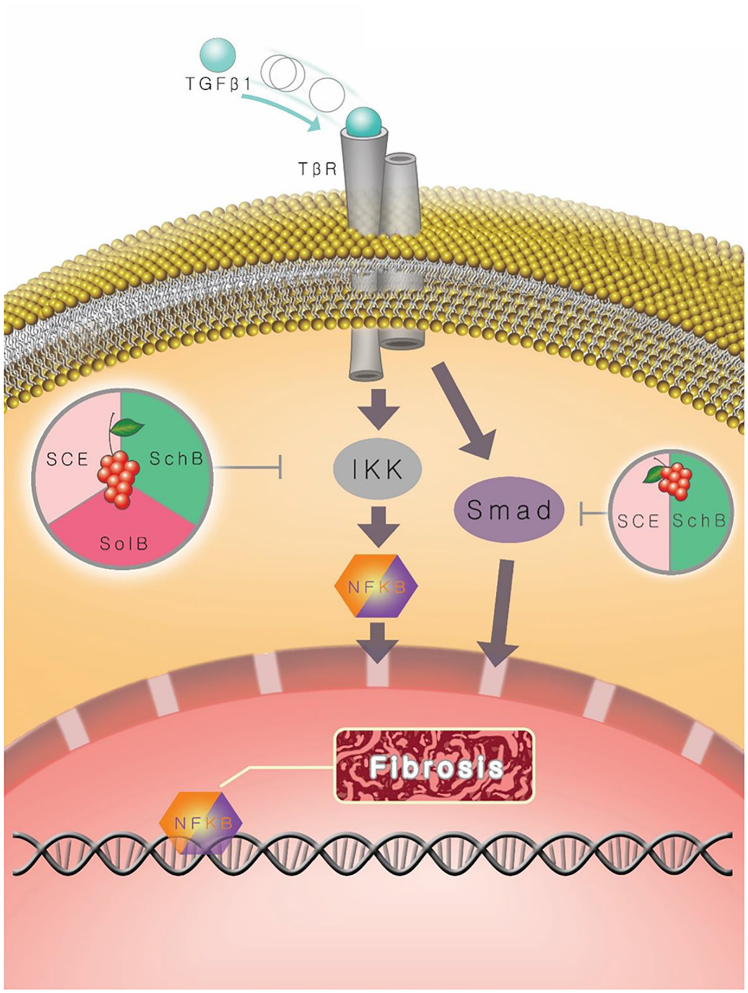

Supplementary Figure 4: Graphical abstract.
